# *Eubacterium rectale* is a potential marker of altered gut microbiota in psoriasis and psoriatic arthritis

**DOI:** 10.1128/spectrum.01154-23

**Published:** 2024-03-05

**Authors:** Yue Xiao, Yiyi Wang, Bangzhuo Tong, Yuanxia Gu, Xingli Zhou, Ning Zhu, Xiaomin Xu, Xiaochen Yin, Yan Kou, Yan Tan, Jincheng Wang, Wei Li

**Affiliations:** 1 Department of Dermatology and Venereology, West China Hospital, Sichuan University, Chengdu, Sichuan, China; 2 Xbiome, Shenzhen, China; Huazhong University of Science and Technology, China

**Keywords:** *Eubacterium rectale*, gut microbiota, metagenomics, psoriasis, psoriatic arthritis

## Abstract

Previous studies have profiled the gut microbiota among psoriatic patients compared to that among healthy individuals. However, a comprehensive understanding of the magnitude, direction, and detailed compositional and functional profiles remains limited. Additionally, research exploring the gut microbiota in the context of both plaque psoriasis (PsO) and psoriatic arthritis (PsA) is lacking. To assess the taxonomic and functional characteristics of the gut microbiota in PsO and PsA patients and investigate potential links between the gut microbiota and disease pathogenesis. We collected fecal samples from 70 psoriatic patients (44 PsO and 26 PsA) and 25 age- and gender-matched healthy controls (HC) and employed deep metagenomic sequencing to characterize their gut microbiota. We noted significant alternations in the gut microbiota compositions of both PsO and PsA patients compared to those of HC. Despite limited effect sizes in alpha diversity (12.3% reduction of microbial richness but unchanged evenness in psoriatic patients) and beta diversity (disease accounts for 3.5% of total variations), we consistently observed substantial reductions of *Eubacterium rectale* in both PsO and PsA patients, with PsA patients exhibiting even lower levels of *E. rectale* than PsO patients. Additionally, two *Alistipes* species were also depleted in psoriatic patients. These microorganisms are known to play crucial roles in carbohydrate metabolism pathways, mainly producing short-chain fatty acids with anti-inflammatory effects. Overall, our observations supplemented the profiling of altered gut microbiota in patients with PsO and PsA at the species level and described a link between the dominant short-chain fatty acid-producing bacterial species and systemic immunity in psoriatic patients.

**IMPORTANCE:**

In this observational clinical study with sufficient sample size and metagenomic sequencing to profile the gut microbiota, we identified consistent signals of the depleted abundance of *Eubacterium rectale* and related functional genes among psoriatic patients, including those with psoriatic arthritis. *E. rectale* may serve as an ecologically important functional unit in the gut microbiota, holding potential as a diagnostic marker and target for therapeutic interventions to achieve lasting effects. Our findings provide comprehensive gut microbiota profiling in psoriasis, resolving previous contradictions and generating new hypotheses for further investigation. These insights may significantly impact psoriasis management and related conditions.

## INTRODUCTION

The gut microbiota is critical in the defense against pathogens, metabolism, and maintaining barrier functions (1, 2). Their immunomodulatory potential on distant organs has been increasingly recognized (3, 4): plaque psoriasis (PsO), psoriatic arthritis (PsA) (5), rheumatologic arthritis (6), nonalcoholic fatty liver disease (NAFLD) (7), and Alzheimer’s disease (8) have all been associated with the dysbiosis of gut microbiota. The concept of the gut–organ axis has been proposed to encapsulate this type of correlation (4).

Psoriasis is a common, chronic, immune-related cutaneous disease with many comorbidities, such as PsA, metabolic syndrome, and NAFLD (9). The pathogenesis is complex and not fully elucidated, involving both environmental and endogenous factors, among which the T-helper (Th)−17/Interleukin (IL)−23 axis has been established as its key immunological mechanism, which is the basis of biologics treatment (10–12). Notably, multiple lines of evidence have suggested that the gut microbiota is critical in maintaining host immune homeostasis, especially affecting the balance of regulatory T cells (Tregs) and effector T cells such as Th1, Th2, and Th17 (13). In particular, one study on imiquimod-induced mouse models shows that gut microbiota significantly affects the manifestation of the psoriatic phenotype through a Th17-mediated T-cell response; notably, germ-free mice or conventionally housed mice treated with antibiotics exhibited reduced psoriatic skin inflammation (14). This evidence supports the hypothesis that altered gut microbiota may contribute to psoriasis pathogenesis by modulating the immune response (15).

Previous studies have profiled the taxonomic and functional characteristics of the gut microbiota in patients with psoriasis based on amplicon or metagenomic sequencing (16–20). Chen et al. identified an imbalance between the phylum *Firmicutes* and *Bacteroides* in psoriasis patients through 16S sequencing and suggested the overrepresentation of bacterial chemotaxis and carbohydrate transport in their gut microbiota (16). Todberg et al. reported lower community diversity in the gut microbiota of psoriatic patients, and the severity of the condition was correlated with these changes using metagenomic sequencing (20). However, due to limitations in cohort sizes and technical challenges, the effect size, direction, and specific disease-associated components of the altered gut microbiota remain unclear. Moreover, research on gut microbial characteristics in PsA is lacking despite its potential role in shaping this distinct phenotype. In this study, we performed a cross-sectional profiling of PsO, PsA, and age- and gender-matched healthy controls (HC) using metagenomic sequencing. We carefully examined the composition and function of the gut microbiota in these patients compared with that in HC. In addition, as previous studies have shown contradictory results on the impact of biologic treatment on the gut microbiota (21, 22), we explored the association of gut microbiota with the therapeutic effectiveness among those patients who received biologics (TNF-α inhibitors or IL-17 inhibitors).

## RESULTS

### Patients, sampling, and sequencing characteristics

We enrolled 95 participants in this study, consisting of 44 patients with PsO, 26 patients with PsA, and 25 HC. The baseline demographic information and clinical characteristics of these three groups are summarized in Table 1. The results of laboratory tests were also collected (Table S1).

**TABLE 1:** The baseline demographic and clinical characteristics of participants^*d*^

	Healthy control (*N* = 25)	Plaque psoriasis (*N* = 44)	Psoriatic arthritis (*N* = 26)
Age, Mean (SD)	33.2 (11.8)	33.0 (9.0)	43.2 (9.3)
Gender, Female, N (%)	15 (60%)	20 (45%)	8 (31%)
Height (cm), Mean (SD)	166.3 (7.7)	166.3 (8.0)	168.4 (10.1)
Weight (kg), Mean (SD)	59.4 (8.6)	67.7 (14.2)	68.1 (14.5)
BMI, Mean (SD)	21.4 (2.2)	24.3 (3.7)	23.8 (3.8)
Smoking history, N (%)^*a*^	4 (16%)	11 (25%)	9 (35%)
Drinking history, N (%)^*b*^	6 (24%)	10 (23%)	8 (31%)
PASI, Mean (SD)		5.8 (3.6)	10.4 (13.1)
BSA, Mean (SD)		6.2 (4.8)	14.0 (21.7)
High blood pressure, N (%)		0 (0%)	4 (15%)
Diabetes, N (%)		1(2.3%)	0 (0%)
Cardiovascular disease, N (%)		0 (0%)	1 (3.8%)
Immune rheumatism, N (%)		0 (0%)	0 (0%)
Fatty liver, N (%)^*c*^		12 (55%)	8 (57%)
Liver stiffness, Mean (SD)		5.13 (0.83)	5.54 (1.24)

^*a*^ Smoking history contains current and former smokers.

^*b*^ Drinking history including current and former drinkers.

^*c*^ Among patients with available data (22 out of 44 plaque psoriasis patients and 12 out of 26 psoriatic psoriasis patients completed liver stiffness examination).

^*d*^ BMI, body mass index; BSA, body surface area; PASI, psoriasis area and severity; SD, standard deviation.

A total of 96 samples, including one internal quality control (QC) sample, were sequenced using the Illumina NovaSeq PE150 platform. On average, 42.3 (standard deviation, ±1.9) million raw paired-end reads were obtained, with 39.5 (±1.8) million (~11.9 ± 0.6 Gb/sample) after a stringent quality trimming process, suggesting excellent sequencing quality. The analysis of the internal QC sample meets our pre-defined QC criteria.

### Alternations of the compositions and functional profiles of the gut microbiota in PsO and PsA

**Fig 1:**
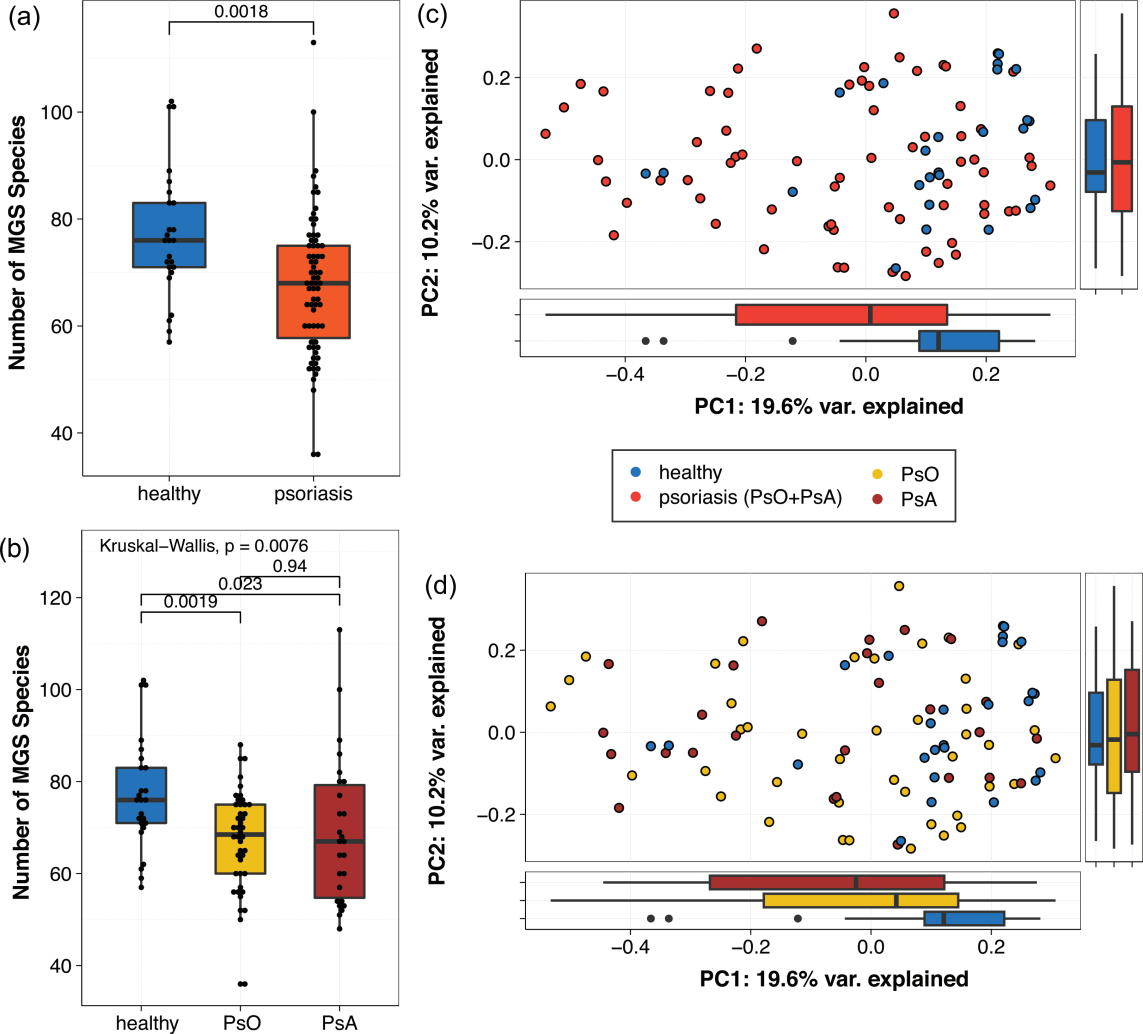
Alternations in gut microbiome among patients compared to age-matched healthy controls. (a, b) Boxplot of the number of metagenomics-inferred bacterial species (MGS species) in each sample by (a) comparing psoriatic patients (plaque psoriasis (PsO) and psoriatic arthritis (PsA) together) to healthy controls, the *P*-value is provided by the Wilcoxon rank-sum test; and (b) comparing PsO, PsA, and healthy controls, the *P*-value is provided by a Kruskal–Wallis test with a post hoc Conover test. (c, d) Principle coordinate analysis (PCoA) based on Bray–Curtis distance inferred from the MGS species profile. The main scatter plot represents the coordinates of each sample; boxplots on the side represent the distribution of either PC1 or PC2 coordinates in each group, with (c) comparing psoriatic patients to healthy controls and (d) comparing PsO, PsA, and healthy controls.

We observed decreases in the gut bacterial species count in samples from patients with PsO and PsA together (Fig. 1a) or separately (Fig. 1b), compared to HC; the extent of these decreases is approximately 12%, which indicates a medium effect size (Cohen’s d = 0.73). The Shannon diversity index of species compositions remains essentially the same between psoriatic patients (PsO and PsA) and HC (Fig. S1). The permutational analysis of variance (PERMANOVA) test (based on Bray–Curtis dissimilarities of species compositions) after correcting for other covariates (i.e., age, gender, BMI) also revealed a significantly altered gut microbiome in psoriatic patients compared to HC for both disease groups together (*P* < 0.001), with an approximate effect size (PERMANOVA R^2^) of 3.6% (Fig. 1c), or separately (*P* < 0.001 and R^2^ = 4.5% for PsO vs HC, *P* < 0.001 and R^2^ = 5.6% for PsA vs HC, Fig. 1d). No significant community-level differences were observed in the gut microbiota between PsO and PsA.

We used KEGG pathway maps to profile the microbial functions in the gut microbiota and found significant differences between disease groups (together or separately to HC) with effect sizes ranging from 4.3% to 7.0% (Fig. S2). Again, no significant differences were found in KEGG profiles comparing PsO to PsA patients (*P* = 0.149).

In addition, when stratifying the patients using PASI scores as mild (PASI <3), moderate (3 ≤ PASI ≤ 10), and severe (PASI＞10), no significant difference was observed, regardless of whether PsO and PsA were analyzed separately (Fig. S3) or together (results not shown). Contrary to previous research (20), these results may suggest that the severity of psoriasis does not alter the gut microbiome. Additionally, we did not observe a correlation between the fatty liver, a common comorbidity previously shown to modify the gut microbiome, and the gut microbiome among psoriasis patients (Fig. S4).

### *Eubacterium rectale* and other species are highly depleted in PsO and PsA

To identify key bacterial species contributing to the alternations in the gut microbiota in psoriatic patients, we conducted a preliminary analysis using Principal Coordinate Analysis (PCoA) coordinates. This analysis revealed a strong correlation between the relative abundance of *E. rectale* and PC1 (rho = 0.720, *P* < 0.001). In the species composition plot (Fig. S5), samples with reduced levels of *E. rectale* were predominantly from PsO or PsA patients.

**Fig 2:**
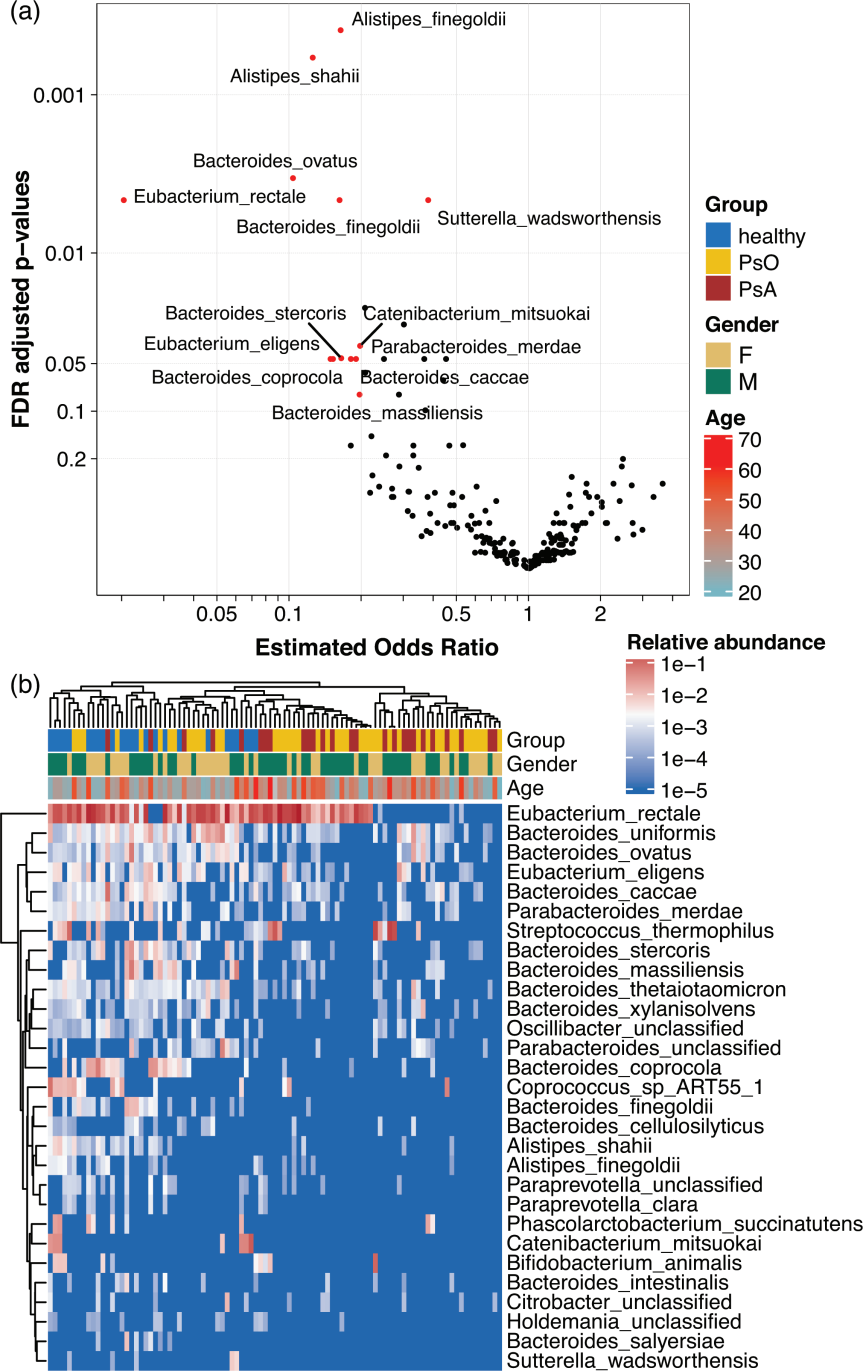
Differential abundance metagenomics-inferred bacterial species (MGS species) identified using logistic regression. (a) The volcano plots show the comparison of patients with psoriasis to healthy controls. An estimated odds ratio (OR)>1 indicates psoriatic patients are enriched and vice versa. MGS species with an adjusted *P* ≤ 0.01 or a revised *P* ≤ 0.1 and an OR <0.2 are highlighted as red dots and labeled. (b) Heatmap of relative abundance of MGS species with an adjusted *P* ≤ 0.2. Column and row dendrograms are plotted using pairwise Euclidian distances. PsO indicates plaque psoriasis, and PsA indicates psoriatic arthritis.

Using a logistic regression-based framework (23), we performed the differential abundance analysis, which confirmed that *E. rectale* was highly depleted in PsO and PsA, with an FDR-adjusted *P* (*P*_adj_) <0.01 and an estimated odds ratio of 0.021 (Fig. 2), almost 50 times more likely to appear in healthy individuals than in psoriasis patients. In addition to *E. rectale*, two *Alistipes* species, *A. finegoldii* and *A. shahii*, were also highly depleted (*P*_adj_ <0.001) in psoriasis patients; all these species were more than five times more likely to present in HC. Several *Bacteroides* species, including *B. ovatus*, *B. finegoldii*, *B. stercoris*, *B. caccae*, and *B. coprocola*, were enriched in HC with a low *P*_adj_ (Fig. 2). The findings were further confirmed using a linear discriminant analysis effect size (LEfSe) (Fig. S6). While comparing PsO to PsA patients, no species showed significant differential abundance after FDR adjustment; however, *E. rectale* appeared to be further reduced in PsA patients (with an OR of nearly 0.1 and an unadjusted *P*-value of 0.059, Fig. S7a)

**Fig 3:**
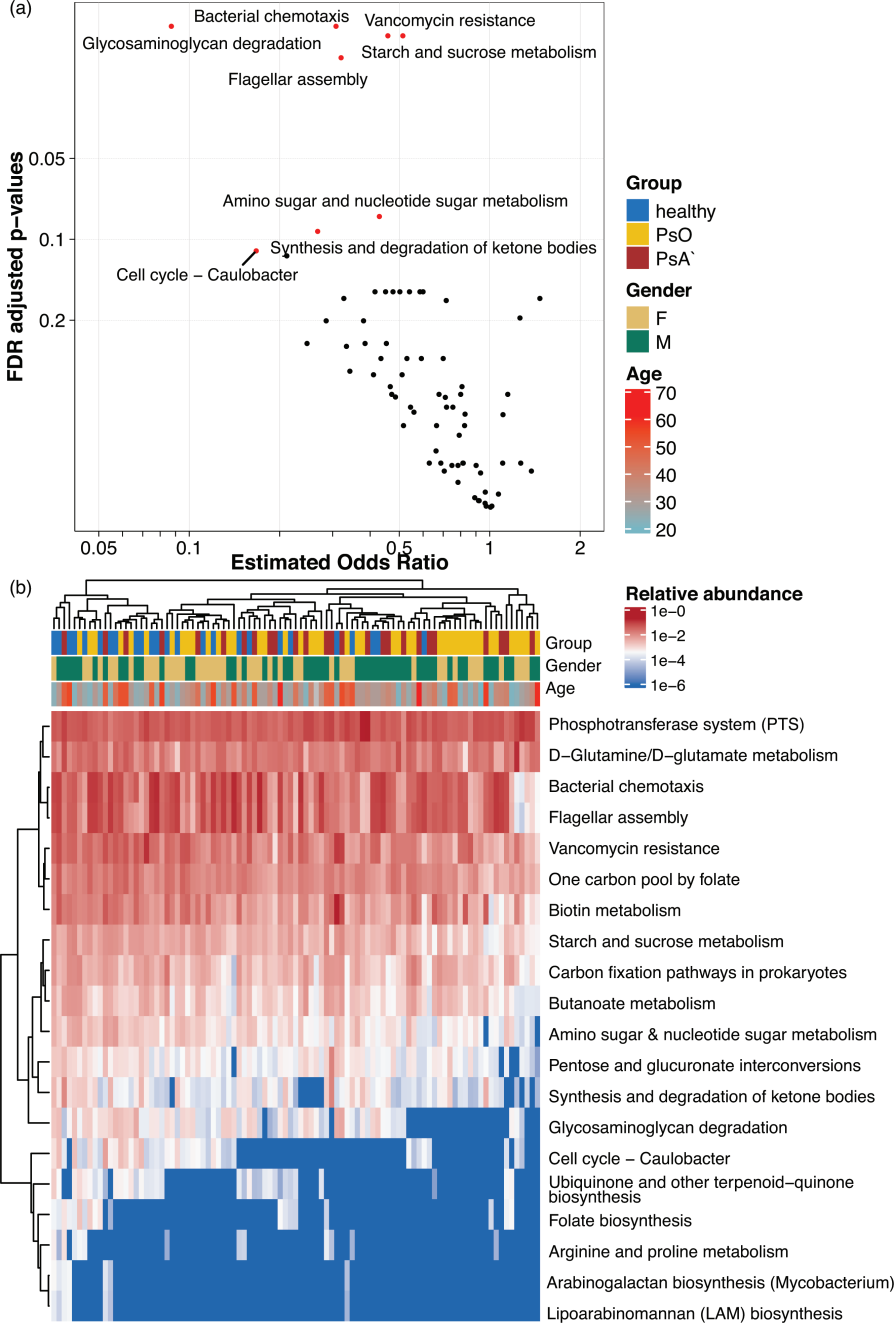
Differential abundance KEGG pathways identified using logistic regression. (a) The volcano plots show the comparison of psoriatic patients to healthy controls. An OR >1 indicates psoriatic patients are enriched and vice versa. Pathways with an adjusted *P* ≤ 0.1 or an adjusted *P* < 0.2 and an OR <0.2 are highlighted as red dots and labeled. (b) Heatmap of relative abundance of KEGG pathways with an adjusted *P* < 0.2. Column and row dendrograms are plotted using pairwise Euclidian distances. PsO indicates plaque psoriasis, and PsA indicates psoriatic arthritis.

When examining the KEGG pathways, starch and sucrose metabolism (OR_ref = healthy_ =0.514, adjusted *P*-value (*P*_adj_) = 0.018), bacterial chemotaxis (OR = 0.308, *P*_adj_ = 0.016), flagellar assembly (OR = 0.320, *P*_adj_ = 0.021), and butanoate metabolism (OR = 0.586, *P* = 0.028/*P*_adj_ = 0.157) were significantly depleted in psoriasis patients compared to that in HC (Fig. 3), to which *E. rectale* was the major contributor (Fig. S8). Meanwhile, only one KEGG pathway, synthesis, and degradation of ketone bodies (KEGG pathway 00072) was highly and significantly depleted in PsA compared to that in PsO patients (OR = 0.22, *P*_adj_ = 0.044), suggesting this pathway is nearly five times more likely to be present in PsO (Fig. S9).

Because, typically, only a small portion of metagenomic sequences could be mapped to fully constructed KEGG pathway maps (in our case, ~20% of all sequences, Fig. S10a), we explored the functional profiles of the gut microbiome at gene family and gene ortholog levels, greatly increasing the sequence usage. About 65% of sequences could be mapped to a KEGG gene family (Fig. S10a), corresponding to 3,075,698 gene families, among which 1,077,614 appeared in more than 80% of samples (prevalent features) and were therefore tested. A total of 765 gene families were significantly different, with more than five-fold changes (OR ≤0.2 or ≥5), all enriched in HC compared to all psoriatic patients (Fig. S10b). Nearly all these genes (717 genes) were from *E. rectale* strains. When the gene families were grouped as KEGG orthologs (KOs), 7,532 KOs were identified, and 55 prevalent KOs were highly significant after FDR correction with >5-fold changes, all enriched in HC (Fig. S10c). Most of these KOs were associated with cellular processing (KEGG category 09140, 17 KOs), environmental information processing (KEGG 09130, 11 KOs), and carbohydrate metabolism (KEGG 09101, 10 KOs). When examining the species’ contributions to these KOs, we again found that *E. rectale* was the dominant (if not the sole) contributor. In addition, signatures of *Alistipes* and *Bacteroides* can be found in several KOs; however, there are much fewer dominant contributions (Table S2). Many KOs also had a high effect size (OR ≤0.2 or ≥5) when comparing PsO and PsA, but their *P*-values were rather high before or after FDR adjustments (Fig. S7b). Therefore, no KO can be identified as differential in this comparison. Given the consistent signal of *E. rectale* in both relative abundances and functional profiles, we conducted tests to determine whether the relative abundance of *E. rectale* could effectively distinguish psoriatic patients from HC. Our analysis yielded a moderately good Area Under the Curve scores, ranging from 0.74 to 0.77 in the receiver operating characteristic curve analysis (Fig. S11). These results indicate that *E. rectale* shows promise as a potential diagnostic marker for differentiating psoriatic patients from healthy individuals.

To further validate the *E. rectale* signal, we assembled metagenomic sequences to recover the genomic bins in each sample, arguably the most sensitive method for assessing microbial community compositions. A total of 1,187 non-redundant genomic bins were obtained, with a range of 52 to 270 bins per sample, among which bins identified as *E. rectale* (*Agathobacter rectalis* in GTDB taxonomy) were recovered in 62 samples. Comparisons of the estimated relative abundance of these bins revealed significantly higher levels of *E. rectale* bins in healthy individuals compared to psoriatic patients (Fig. S13a). Furthermore, a nested PCR protocol described by Kageyama and Benno (24) was employed to further confirm our findings. As shown in Fig. S13b, we successfully identified *E. rectale*-specific bands in 70 samples, with 24 out of 25 samples from healthy individuals exhibiting clear bands. This pattern highly correlates with the abundance of *E. rectale*-specific bins, providing additional validation for the enrichment of *E. rectale* in healthy individuals.

### The baseline gut microbiota is not correlated with the effectiveness of biologics

A sub-cohort of 22 psoriasis patients (13 PsO and 9 PsA) who were treated with either Secukinumab or Adalimumab were followed across their treatment durations, and the association between their baseline microbiota and the treatment effectiveness was explored. Given the nature and common regimen of the two treatments, we used PASI75 (a PASI score exceeding 75%) to mark the effectiveness of Adalimumab (Ada group) and PASI90 for Secukinumab (Sec group) and 32 weeks post-treatment as the evaluation endpoint. A total of 12 patients (six PsO and six PsA patients) reached PASI75 by 32 weeks, but no significant differences were found in the baseline gut microbiota comparing the 12 responders to the ten non-responders (PERMANOVA *P* = 0.349 after correcting for PsO vs PsA and biologics), suggesting the effectiveness of biologics may not be correlated with their baseline gut microbiota. Meanwhile, despite significant differences in effectiveness between the two biologics (log-rank sum test, P = 0.002, Fig. S12), no significant differences were found in PsO to PsA (*P* = 1.000).

## DISCUSSION

In this study, we performed metagenomic sequencing analysis to explore the altered gut microbiota among a large cohort of patients with psoriasis compared to HC. We demonstrated a noticeable reduction of gut microbial diversity in both PsO and PsA and the reduction of several species that may have probiotic effects; several microbial functional pathways, such as butanoate metabolism, may be related to the lost species.

Consistent with previous findings, we observed significant changes in microbial compositions among psoriatic patients: a reduction in the richness but not overall evenness (as measured by the Shannon index) and a significantly altered community-measured beta diversity. However, the effect sizes of these alternations were limited, ranging from small to medium [based on the guidelines summarized in (25)]. These results are consistent with previous reports after reviewing the size of reported differences in the respective studies (5, 17, 19, 20) and may also explain an opposite direction, as reported by Shapiro et al., because a small effect is susceptible to the effects of covariates, technical errors, or random errors (18).

In addition to community-level alternations among psoriatic patients, we identified several differentially abundant features that may have potential health implications. We identified consistent signals from *E. rectale. E. rectale*, recently proposed as an *Agathobacter rectalis* comb. nov. under the family of *Lachnospiraceae*, is a gram-positive, non-spore-forming, obligately anaerobic, monoflagellated bacterium (26). In addition to its well-known ability to produce butyrate, it can also produce acetate, hydrogen, and lactate (26). Furthermore, the abundance of KEGG KOs and functional pathways associated with this species were highly depleted in psoriasis patients, making a solid case that *E. rectale* could be the central piece of a microbial ecological functional unit, which may have health implications. Depletion of the butyrate-producing *Eubacterium* or *E. rectale* has also been reported in other diseases: Takahashi and colleagues observed a reduced relative abundance of *Eubacterium* genera in patients with Crohn’s disease (27), and Vermeiren et al. reported a reduced diversity of fecal *Clostridium coccoides*/*E. rectale* species in patients with ulcerative colitis (28). Loomba et al. found that *E. rectale* was abundant in mild or moderate NAFLD, whereas *Escherichia coli* was the dominant species in advanced fibrosis (7). In parallel, these results suggest that the gut microbiota dysbiosis, particularly the depleted levels of *E. rectale* in the disease cohorts, may be related to the damaged mucus barrier and the progression of liver fibrosis. Additionally, we found that *A. finegoldii* and *A. shahii* species were significantly reduced, which is consistent with previous findings in psoriasis and a cohort of patients with Crohn’s disease (5, 19, 29). It is interesting to note that the species profiles depleted in psoriasis were somehow similar to those of inflammatory bowel disease (IBD) patients, in whom all the above-mentioned bacterial species are thought to play a protective role.

Most of the species depleted in psoriasis in this study were possible producers of short-chain fatty acids (SCFAs), which is supported by our functional analysis results, as starch and sucrose metabolism, bacterial chemotaxis, and butanoate metabolism were all depleted in psoriasis patients. In addition to the butyrate-producing *E. rectale*, Alistipes, especially the *A. shahii* species, have been shown to elevate propionate levels in the gut (30). SCFA is a preferred fuel for the colonocytes and is essential for maintaining the epithelial barrier (31, 32). Propionate is associated with gluconeogenesis in the liver and may have potential beneficial effects (32, 33). Meanwhile, these SCFA-producing organisms may exert their protective effects by regulating the immune system. It has been shown that SCFAs could induce the differentiation of effector T cells and Tregs and suppress the polarization of Th17 cells (32, 34). These immunomodulatory effects of gut microbiota have been demonstrated by fecal microbiota transplantation (FMT) in animal models and even humans. The imiquimod-induced psoriasis-like mouse model, which received FMT from healthy donors with a *Lactobacillus* supplement, showed a protective effect against Treg/Th17 imbalance (35). Moreover, clinical trials have shown that FMT also appears effective in treating IBD patients (36).

Intriguingly, we did not observe a significant correlation between the baseline gut microbiota and the clinical prognosis of MAB treatments. Among the differentially abundant species identified by comparing psoriasis patients with HC, none showed a significant difference between responders and non-responders. Interestingly, *E. rectale* appeared to have a much lower relative abundance in responders than in non-responders, although not significantly (*P* = 0.17, median abundance_responders_ = 0.68% vs median abundance_non-responders_ = 10.39%). It can be hypothesized that biologics would work better in psoriasis patients depleted with *E. rectale* functional groups.

Overall, our observations provide further evidence of detailed alternations in the gut microbiota at the species level in one of the larger cohorts of psoriasis patients, assess the magnitude and aspects of these alternations, and provide comprehensive evidence of the altered species and functions. However, there are several limitations due to the nature of this exploratory study, as we can only speculate on the potential association between the altered gut microbiota and psoriasis rather than reaching a more plausible hypothesis on the causal relationship. Furthermore, we did not have access to the metabolomic profile of either host or microbial communities to directly confirm the role of SCFAs in psoriasis, although our results strongly suggest a role for SCFAs. Nevertheless, we have generated several hypotheses that should be tested in longitudinal observational studies or even randomized clinical trials, such as the depletion of possible probiotic bacterial species, including *E. rectale, A. finegoldii,* and *A. shahii*, or the therapeutic potential of SCFAs supplementation or the live bacterial strains.

## MATERIALS AND METHODS

### Participants

This study was conducted in compliance with the Declaration of Helsinki and was approved by the biomedical research ethics committee of the West China Hospital of Sichuan University (Approval number: 2020–234). Psoriasis patients were enrolled in the Department of Dermatology & Venereology of West China Hospital, Sichuan University, from September 2020 to March 2021. Healthy subjects from the same region were recruited as controls. All of them were informed of the purpose of this study and signed the consent form before the process began. The number of subjects to include for each group was calculated using the micropower algorithm (37): assuming an effect size of 4% comparing the microbial community differences (based on weighted beta diversity measurement) between disease groups and healthy subjects, 20 subjects per group were required to afford a power of 0.90 and therefore were originally targeted.

### Inclusion and exclusion criteria

The inclusion criteria for the participants required 18–65 years. The psoriasis group consisted of diagnosed plaque psoriasis patients, then into subgroups by screening for the presence of PsA: psoriasis patients who met the Classification Criteria for Psoriatic Arthritis (CASPAR) were included in the PsA subgroup, while other patients were in the PsO subgroup. HC from the same geographic regions were matched by age and gender. The exclusion criteria applied to all the participants in this study, including during pregnancy and lactation, who used systematic medication (such as immunosuppressants, biologics, antibiotics, and probiotics) in the past three months with other immune-related diseases (inflammatory bowel disease), severe infectious diseases, malignancies combined, and received gastric surgery.

### Sample collection and processing

Fresh fecal samples were collected from participants at the first visit; approximately 30–50 g were sampled in a sterile container and stored at −20℃ immediately. These samples were transferred to −80℃ within 6 h and stored until DNA extraction. Microbial DNA was extracted using the Qiagen DNeasy 96 PowerSoil Pro QIAcube HT kits according to the manufacturer’s protocol. Extracted DNAs were delivered to a commercial genomic sequencing service lab, Novogene, for library preparation using the NEBNext Ultra II DNA kit and sequencing using Illumina NovaSeq platforms.

A nested PCR protocol was adopted to further validate the presence of *E. rectale* (24). Briefly, a pair of full-length, universal 16S primers were used to amplify the bacteria DNAs (Forward: 27F 5′-AGAGTTTGATCCTGGCTCAG-3′, Reverse: 1492R 5′-GGTTACCTTGTTACGACTT-3′); 10 ng of extracted DNA were used for the amplification with the following conditions: one cycle of pre-denaturation at 95°C for 3 minutes, 25 cycles of denaturation at 95°C for 20 seconds, annealing at 55°C for 30 seconds, elongation at 72°C for 30 seconds, and one cycle of post-elongation at 72°C for 5 minutes. Following the generation of primary amplicons, the *E. rectale*-specific amplicons were generated using primer pair (Forward: rec-F:5′-CATIGCTICTCGGTGCCGTC-3′, Reverse: rec-R:5′-ATITGCTCGGCTTCACAGCT-3′) and 2 µL of primary amplification products, with the following conditions: one cycle of 95°C for 3 minutes, 20 cycles of denaturation at 95°C for 20 seconds, annealing at 65°C for 15 seconds, elongation at 72°C for 30 seconds, and one cycle of post-elongation at 72°C for 5 minutes. The resulting amplicons were tested on an electrophoresis gel system, and an amplicon of roughly 440 bps was expected.

### Metagenomic sequencing analysis

The PE150 reads from Illumina NovaSeq were obtained and processed using our in-house pipeline, which consists of the following tools: fastp v0.20 (38) was used to trim the sequencing and PCR adapters and filter short sequences (<50 bp), followed by kneaddata v0.10 (39) to trim the low-quality reads (a sliding window of 4 and an average quality score of 20 were used) and decontaminate against the hg19 human genome; humann2 v2.8.1 (40) was used to generate taxonomy profiles and functional profiles with the KEGG database (41). Alpha diversities based on taxonomy profile were obtained in the form of metagenomic species count and Shannon index using vegan v2.6.2. Beta diversities of taxonomy or functional profiles were obtained using the Bray–Curtis distance, and PCoA was performed using the cmdscale function in vegan v2.6.2 (42).

Metagenomic assembly was performed with the previously generated clean reads using our in-house pipeline, consisting of the following steps: megahit v1.2.9 (43) was used for assembling, followed by a parallel binning step using concoct v1.1.0 (44), maxbin2 v2.2.7 (45), and metabat2 v2.15 (46), respectively; the binning results were filtered and merged using DAS Tool v1.1.6 (47) with bin completeness over 75%, contamination rate less than 25%, and total length over 500 kb. Merged bins were dereplicated using dRep v2.2.3 (48) and annotated using GTDB-Tk v2.3.2 (49) and GTDB release 202 (50) with the bin counts generated by salmon v0.13.1 (51).

### Statistical analysis

Welch’s two-sample *t*-test was used for tests in the whole blood count and lipid panel. A significance level of 0.05 was set. For microbiome analysis, Wilcoxon’s rank sum test or Kruskal–Wallis tests were performed for univariate comparison (e.g., tests for alpha diversities), with the Conover–Iman test (52) as the post hoc when necessary. A comparison based on beta diversities was performed using Permutational Multivariate Analysis of Variance (PERMANOVA) (53), adjusted for covariates using vegan v2.6.2 (42). The R^2^ calculated during the tests was used as an approximate measurement of effect sizes.

For the abundant differential features, a logistic regression-based framework was applied (23). After prevalence filtering (usually a cut-off of 0.2 is used, except for the KEGG gene family and KOs tests due to the too many features, in which case a cut-off of 0.8 is used), the relative abundance is modeled as the probability of the features to be presented in the samples and converted by a logit function and input as the response variable in the logistic regression. The coefficients obtained can be converted to an odds ratio estimate after an exponential transformation. The *P*-values obtained were subjected to FDR adjustment. A combination of adjusted *P*-values (statistical significance, typically <0.2) and odds ratios (a measure of effect size, typically <0.2 or >5) are used to infer differential abundant features. Specific cut-off values were selected given the number of tests and overall expectations of the effect size and were noted in the results whenever necessary. The linear discriminant analysis effect size (LEfSe) (54) was performed through an R implementation lefser (55) to validate the fatty liver disease (NAFLD) findings. The mean total estimated clade counts for each sample from MetaPhlAn2 outputs (through Humann2) were used as the normalization factor; 0.05 was used for initial Wilcoxon and Kruskal–Wallis test screening; and an LDA score >2 was used to select the final significant features.

The cumulative incidence of responding to biologic treatments was evaluated using the ggsurvfit v0.2.1 package (56) and compared using the log-rank sum test available through the survival v3.1.8 package (57). In addition, phyloseq v1.30.0 (58) was extensively used in the analyses. All statistical analyses were done in R v3.6.3 (59). All codes for data analysis and creating illustrations are hosted on Github at https://github.com/ETaSky/xbiome_psoriasis_ms.

## Data Availability

The sequencing data after quality trimming and removing of host sequences that support the findings of this study are openly available in NCBI SRA with accession number PRJNA938297.

## SUPPLEMENTAL MATERIAL

Tables S1 and S2 spectrum.01154-23-s0001.docx Table S1 (complete blood counts and lipid panel from patients) and Table S2 (the file of KEGG orthologs).

Fig. S1 and S2 spectrum.01154-23-s0002.eps Figure S1 (Comparison of Shannon diversity index in gut microbiota) and Figure S2 (Principle coordinate analysis [PCoA] based on Bray–Curtis distance inferred from KEGG pathway maps profile).

Fig. S3 spectrum.01154-23-s0003.eps Comparison of alpha diversities based on PASI categories among patients with psoriasis.

Fig. S4 spectrum.01154-23-s0004.eps Comparison of alpha diversities based on fatty liver diagnosis among psoriatic patients.

Fig. S5 and S6 spectrum.01154-23-s0005.eps Figure S5 (MGS species compositions in each sample; each column represents a sample and is colored by MGS species with >1% of total abundance in each sample) and Figure S6 (differential abundance MGS species identified using LEfSe).

Fig. S7 spectrum.01154-23-s0006.eps Differential abundance analysis comparing psoriatic arthritis to plaque psoriasis patients at (a) MGS species level and (b) KEGG gene orthologs level.

Fig. S8 spectrum.01154-23-s0007.eps Species contribution to the identified KEGG pathways related to *Eubacterium rectale*. Pathway name is annotated in the panels.

Fig. S9 and S10 spectrum.01154-23-s0008.eps Figure S9 (differential abundance analysis comparing patients with psoriasis arthritis to plaque psoriasis in KEGG pathways) and Figure S10 (investigation of the functional profiles based on the KEGG database).

Fig. S11 spectrum.01154-23-s0009.eps Receiver operating characteristic curve analysis of *Eubacterium rectale* for differentiating psoriatic and healthy individuals.

Fig. S12 and S13 spectrum.01154-23-s0010.eps Figure S12 (cumulative incidence of treatment remission among a sub-cohort of patients) and Figure S13 (validation of *Eubacterium rectale*).

ASM does not own the copyrights to Supplemental Material that may be linked to, or accessed through, an article. The authors have granted ASM a non-exclusive, world-wide license to publish the Supplemental Material files. Please contact the corresponding author directly for reuse.
