# Supplementary material for: Eubacterium rectale is a potential marker of altered gut microbiota in psoriasis and psoriatic arthritis
Source: Microbiol Spectr. 2024 Mar 5;12(4):e01154-23. doi: 10.1128/spectrum.01154-23 (PMC10986482; doi:10.1128/spectrum.01154-23)
Supplement: Tables S1 and S2 — Table S1 (complete blood counts and lipid panel from patients) and Table S2 (the file of KEGG orthologs). [file spectrum.01154-23-s0001.docx]

**Table S2.** The file of KEGG orthologs
